# Efficacy and Safety of Apatinib Plus Vinorelbine in Patients With Wild-Type Advanced Non–Small Cell Lung Cancer After Second-Line Treatment Failure: A Nonrandomized Clinical Trial

**DOI:** 10.1001/jamanetworkopen.2020.1226

**Published:** 2020-03-19

**Authors:** Xiangyu Zhang, Yi Xiong, Qing Xia, Fang Wu, Lingli Liu, Yuling Zhou, Liang Zeng, Chunhua Zhou, Chen Xia, Wenjuan Jiang, Dehua Liao, Lili Xiao, Li Liu, Haiyan Yang, Rui Guan, Kunyan Li, Jing Wang, Guang Lei, Yongchang Zhang, Nong Yang

**Affiliations:** 1 Department of Medical Oncology, Lung Cancer and Gastrointestinal Unit, Hunan Cancer Hospital/The Affiliated Cancer Hospital of Xiangya School of Medicine, Central South University, Changsha, China; 2 State Key Laboratory for Oncogenes and Related Genes, Shanghai Cancer Institute, Renji Hospital, Department of Oncology, Shanghai Jiao Tong University School of Medicine, Shanghai, China; 3 Graduate Schools, University of South China, Hengyang, China; 4 Department of Hepatology, Hunan Cancer Hospital, Changsha, China; 5 Department of Pharmacy, Hunan Cancer Hospital, Changsha, China; 6 Center of New Drug Clinical Trials, Hunan Cancer Hospital, Changsha, China; 7 Hunan Clinical Research Center in Gynecologic Cancer, Hunan Cancer Hospital/The Affiliated Cancer Hospital of Xiangya School of Medicine, Central South University, Changsha, China; 8 Department of Experimental Radiation Oncology, University of Texas MD Anderson Cancer Center, Houston

## Abstract

**Importance:**

There is currently no standard treatment strategy for patients with advanced non–small cell lung cancer (NSCLC) without driver gene variation after failure of 2 or more lines of chemotherapy.

**Objective:**

To assess the efficacy and safety of apatinib combined with oral vinorelbine.

**Design, Setting, and Participants:**

This phase 2 prospective nonrandomized clinical trial evaluating the efficacy and safety of apatinib plus vinorelbine recruited patients from Hunan Cancer Center, Hunan, China, from January 1, 2017, to November 30, 2018. Eligible patients were those with wild-type advanced NSCLC whose disease did not respond to at least 2 lines of chemotherapy. Patients were evaluated until December 31, 2019. Data were analyzed from July 2019 to December 2019.

**Intervention:**

Apatinib at an initial dose of 500 mg once daily and oral vinorelbine 60 mg/m^2^ once weekly were administered until disease progression, patient withdrawal, or occurrence of unacceptable toxic effects.

**Main Outcomes and Measures:**

The primary end point was overall response rate. Secondary end points were overall survival, progression-free survival, and safety.

**Results:**

The potential efficacy of apatinib plus vinorelbine was identified using drug susceptibility assay based on 3-dimensional coculture of tumor cells derived from 3 patients with lung adenocarcinoma. Among 30 patients enrolled, the median (range) age was 63 (34-78) years and 18 (60%) were men. Most patients (27 patients [90%]) had stage IV disease, and the median (range) number of prior unsuccessful treatments was 2 (2-5) lines of chemotherapy. Twenty-five patients (83%) completed the treatment, while 5 patients (17%) discontinued treatment owing to intolerable adverse events. The overall response rate was 36.7% (11 patients) and the disease control rate was 76.7% (23 patients). The median progression-free survival was 4.5 (95% CI, 2.4-6.6) months, and the median overall survival was 10.0 (95% CI, 4.8-17.1) months. Hand-foot syndrome was the most common adverse event observed, including grade 3 hand-foot syndrome observed in 5 patients (17%) and grade 4 hand-foot observed in 1 patient (3%). Grade 3 weakness was observed in 1 patient (3%).

**Conclusions and Relevance:**

These findings suggest that apatinib combined with oral vinorelbine is a potentially effective regimen with an acceptable safety profile. This regimen may have potential as a treatment option for patients with wild-type advanced NSCLC whose disease failed at least 2 prior lines of chemotherapy.

**Trial Registration:**

ClinicalTrials.gov Identifier: NCT03652857

## Key Points

**Question:**

What are the safety and efficacy of apatinib plus vinorelbine for patients with wild-type advanced non–small cell lung cancer who are experiencing progression after 2 or more lines of chemotherapy?

**Findings:**

In this phase 2 nonrandomized controlled trial of 30 patients with wild-type advanced non–small cell lung cancer, apatinib plus vinorelbine administered after failure of at least 2 lines of previous chemotherapy regimen was associated with significantly increased overall response rate and prolonged median progression-free survival and overall survival, and they were associated with manageable toxic effects. The potential efficacy of apatinib plus vinorelbine combination was identified using a 3-dimensional coculture platform.

**Meaning:**

These findings suggest that apatinib plus vinorelbine may be an effective and safe regimen as subsequent-line therapy in patients with wild-type advanced non–small cell lung cancer.

## Teaser

This nonrandomized clinical trial examines the efficacy and safety of apatinib plus vinorelbine in patients in China with wild-type advanced non–small cell lung cancer (NCSLC) that was resistant to 2 or more lines of chemotherapy.

## Introduction

Lung cancer is the leading cause of cancer-related death in China and worldwide.^1,2^ In China, approximately 750 000 people are diagnosed with lung cancer annually.^2^ Non–small cell lung cancer (NSCLC) accounts for 85% of lung cancers diagnosed.^1^ First- and second-line chemotherapies provide survival benefit to patients with advanced NSCLC without actionable variations in oncogenic genes, including *EGFR* (OMIM 131550), *ALK* (OMIM 105590), and *ROS1* (OMIM 165020).^3,4,5,6,7,8^ Some chemotherapy regimens, including gemcitabine and docetaxel, have also shown activity as third-line treatments but remain controversial as standard treatments.^9^ Angiogenesis is a hallmark of cancer and is responsible for tumor spread and metastasis.^10,11^ Antiangiogenic therapies, including monoclonal antibodies against *VEGF* (OMIM 192240) and multireceptor tyrosine kinase inhibitors (TKIs), such as VEGF receptor TKIs, as single agents or in combination with other agents such as chemotherapy or targeted therapy, have been shown to be effective therapeutic strategies for NSCLC.^12,13,14^ Apatinib is a novel VEGF receptor 2 TKI with encouraging antitumor activities and tolerable toxic effects in several malignant tumors.^15,16,17^ In a 2018 phase 2 study by Liu et al,^18^ apatinib monotherapy administered as third-line therapy to 34 patients with advanced NSCLC achieved an objective response rate (ORR) of 5.9%, and median progression-free survival (PFS) of 4 months, although the authors did not report the median overall survival. Vinorelbine is a chemotherapeutic agent designed to inhibit microtubules of cancer cells, which leads to cell death.^19^ In a 2018 phase 2 study,^20^ vinorelbine monotherapy administered to 159 patients with advanced NSCLC after multiline treatment failure achieved an ORR of 19.5% with median PFS of 3 months.

The 3-dimensional (3-D) coculture platform has been demonstrated to accurately simulate in vivo tumor microenvironment and tumor in vivo progression events, including angiogenesis, growth, and metastasis, making it an attractive in vitro model for drug screening.^21^ The process of 3-D coculture involves the growth of various cell populations, including cells derived from patient tumor biopsy, within an intricate but well-organized extracellular matrix to simulate a physiological in vivo–like microenvironment by preserving the native signaling pathways, cell-cell and cell-matrix interactions, and cell morphology or phenotypes.^21^

To identify novel treatment strategies for wild-type advanced NSCLC, a 3-D coculture-based drug susceptibility assay was designed. Based on the promising results from this assay, apatinib plus vinorelbine was identified and a prospective nonrandomized clinical trial was initiated to determine the efficacy and safety of this combination in patients with wild-type advanced NSCLC whose disease had progressed on 2 or more lines of prior chemotherapy.

## Methods

This phase 2, single-group, prospective nonrandomized clinical trial was performed at Hunan Cancer Hospital in Hunan, China. The trial was approved by the Research Ethics Board of Hunan Cancer Hospital and performed in accordance with the Declaration of Helsinki.^22^ All patients provided written informed consent. This study is reported following the Consolidated Standards of Reporting Trials (CONSORT) reporting guideline. Full details of the trial protocol are avaiable in Supplement 1.

### In Vitro 3-D Coculture Drug Susceptibility Test

Three patients with wild-type advanced NSCLC whose disease had failed 2 prior lines of treatment were recruited for the preliminary phase of the study. None of these patients had received prior treatment of either vinorelbine or apatinib. Gene variant status of the 3 patients was confirmed with capture-based targeted next-generation sequencing of the needle biopsy specimen of their primary lung tumor.

The 3-D coculture was performed according to published protocol by Fang and colleagues^21^ at the Department of Medical Oncology, Lung Cancer and Gastrointestinal Unit, Hunan Cancer Hospital. The primary lung tumor sample obtained by needle biopsy from the patient was collected in a tube and washed 3-fold with 1× phosphate-buffered saline. Using a disposable scalpel, the tumor sample was dissected into smaller pieces and stored on ice prior to seeding in a 96-well petri dish, which was precoated with 1.5% low–melting temperature agarose and preseeded with a layer of endothelial cells suspended with Geltrex matrix (Thermo Fisher Scientific). Each 3-D culture was allowed to form a tumor cell colony in the presence of endothelial cell growth basal medium-2 growth medium with endothelial cell growth medium-2 endothelial supplement SingleQuots kits (Lonza). The 3-D culture was then layered with culture media containing the inhibitor and observed under a fluorescence microscope after 24 to 48 hours to evaluate drug susceptibility by quantifying cell proliferation.

The regimen that was found to be potentially effective in the in vitro drug susceptibility test was administered to 3 patients for the preliminary stage. The responses of the patients to the regimen were evaluated by computed tomography scans.

### Patient Selection for Prospective Clinical Study

Patients aged 18 to 80 years with advanced NSCLC who experienced disease progression after 2 or more lines of chemotherapy were eligible to take part in the study. A sample size of 30 patients was fixed for the study. Patients whose disease progressed within 6 months of treatment with a non–platinum-containing regimen were also eligible if they were considered platinum resistant based on the last platinum-containing regimen received. Patient inclusion criteria included histologically confirmed adenocarcinoma, adenosquamous carcinoma, or squamous cell carcinoma; for patients with lung adenocarcinoma, driver variations in *EGFR*, *ALK*, and/or *ROS1* were tested by next-generation sequencing (patients with squamous cell lung carcinoma were not required to undergo variation testing); Eastern Cooperative Oncology Group performance status score of 0 to 2; life expectancy of at least 3 months; no prior exposure to vinorelbine or apatinib; adequate bone marrow function (defined as white blood cell count of ≥3000 cells/μL [to convert to ×10^9^ cells per liter, multiply by 0.001], absolute neutrophil count of ≥1500 cells/μL, platelet count of ≥70 ×10^3^ cells/μL [to convert to ×10^9^ cells per liter, multiply by 1], and hemoglobin concentration of ≥8.0 g/dL [to convert to grams per liter, multiply by 10]); adequate liver function (defined as alanine aminotransferase or aspartate aminotransferase levels ≤80 U/L [to convert to microkatals per liter, multiply by 0.0167] and total bilirubin level ≤342.12 mg/dL [to convert to millimoles per liter, multiply by 17.104]); and adequate renal function (defined as creatinine level ≤7930 mg/dL [to convert to millimoles per liter, multiply by 76.25]). Key exclusion criteria included previous exposure to apatinib or vinorelbine, confirmed *EGFR*, *ALK* or *ROS1* driver variations, contraindication of chemotherapy, and women who were pregnant or lactating. Prior exposure to other antiangiogenic agents, including bevacizumab, was not grounds for exclusion.

### Procedures

Our study treatment consisted of oral apatinib administered at an initial dose of 500 mg once daily and oral vinorelbine at a dose of 60 mg/m^2^ once weekly. Dose reductions were planned according to the drug brochures. Dose reescalation was not allowed. Treatment was continued until disease progression, patient withdrawal, or unacceptable toxic effects.

Dose modifications, including dose interruptions and reductions, were allowed for the management of adverse events. One dose reduction was permitted for apatinib (500 mg or 250 mg used on alternate days or 250 mg once daily). For grade 4 nonhematological toxic effects, apatinib was delayed until recovery to grade 1 or better and then resumed with a reduced dose. At the first occurrence of either grade 3 nonhematological or grade 3 or 4 hematological toxic effects, apatinib was delayed until recovery to grade 1 or below for nonhematological toxic effects and grade 2 or below for hematological toxic effects, then treatment was resumed at the standard dose. After the second occurrence of either grade 3 nonhematological or grade 3 or 4 hematological toxic effects, patients who had dose reduction (to either 500 mg or 250 mg used on alternate days or 250 mg once daily) were treated depending on the dose level they were receiving when the toxic effects occurred. For any grade 3 or 4 toxic effects (except hypertension, hand-foot syndrome, and proteinuria), oral vinorelbine was withheld until resolution to grade 2 or less for hematological toxic effects or grade 1 or less for nonhematological toxic effects, then treatment was resumed with a reduced dose. Dose was reduced for intolerable grade 2 adverse events as necessary.

Measurable disease was assessed and documented before initiating treatment. Patients were examined after the first month of receiving the regimen. Radiological assessments of target and nontarget lesions were performed every 8 weeks by computed tomography scan during the treatment phase until confirmation of disease progression. During follow-up, patients underwent physical examinations that included 12-lead electrocardiogram examination, vital signs testing, routine urine examination, and blood testing, including hematological testing, serum chemistry testing, and testing for biomarkers associated with tumors. Adverse events were graded according to Common Terminology Criteria for Adverse Events version 4.03 and monitored on a weekly basis for the first month, then at least once every 4 weeks until disease progression or last follow-up date of December 31, 2019.

### Outcomes

The primary end point was the proportion of patients who achieved overall response (ie, complete or partial). Secondary end points were PFS (measured from the treatment start date until disease progression or death), overall survival (measured from treatment start date until death), and safety. Objective responses were determined according to Response Evaluation Criteria in Solid Tumors version 1.1. Disease control rate was defined as the proportion of patients with complete response, partial response, and stable disease.

### Statistical Analysis

All statistical analyses were conducted using SPSS statistical software version 22.0 (IBM Corp). The proportions of responders were calculated with 95% CIs using the Clopper-Pearson method. The Kaplan-Meier method was used to estimate the median durations of response and PFS with corresponding 95% CIs. We only analyzed data that were collected by the cutoff date of December 31, 2019. Data analysis was conducted from July 2019 to December 2019.

## Results

### 3-D Coculture Platform

Three male patients who were formerly smokers and who had wild-type advanced lung adenocarcinoma after failure of 2 previous lines of treatment were included in this study (eTable 2 in Supplement 2). Primary lung tumor biopsy samples were obtained, cocultured, and used to perform drug screening of a total of 49 single and combination drugs (eFigure 1 and eTable 1 in Supplement 2). According to the results of the screening using the coculture platform with patient-derived tumor cells, the combination of apatinib and vinorelbine was potentially effective.

Patient 1 had undergone at least 2 prior platinum-based doublet chemotherapy regimens combined with pemetrexed and paclitaxel (eFigure 2A in Supplement 2) and had a confirmed diagnosis of lung adenocarcinoma based on the pathological examination and immunohistochemistry (eFigure 2B in Supplement 2). The results of 3-D in vitro drug susceptibility testing showed that the effects of either apatinib or vinorelbine as single agents, as well as paclitaxel and pemetrexed, were not satisfactory, while the combination of apatinib and vinorelbine had the best inhibitory association with tumor cell proliferation (eFigure 2C and D in Supplement 2). Based on the promising results from the 3-D coculture assay, the patient was administered with apatinib and oral vinorelbine. Review of this patient’s computed tomography results showed a significant reduction in primary lesions, evaluated as partial response, after 2 months of treatment with apatinib plus vinorelbine (eFigure 2E in Supplement 2). This patient’s response remained durable as of December 31, 2019, after 45 months of therapy. Similarly, patients 2 and 3 also had received diagnoses of lung adenocarcinoma and responded to apatinib and vinorelbine therapy at the third-line setting, which lasted for 12 months for patient 2 and 15 months for patient 3.

### Patient Characteristics and Treatment

Between January 1, 2017, and November 30, 2018, a total of 32 patients with advanced NSCLC were screened for eligibility. Of them, 30 patients were enrolled in the cohort (eFigure 3 in Supplement 2). Table 1 summarizes the baseline demographic characteristics of the study cohort. The median (range) age of the cohort was 63 (34-78) years, and 18 patients (60%) were men. Nineteen patients (63%) had received diagnoses of adenocarcinoma, all of whom underwent variation testing for *EGFR*, *ALK*, and *ROS1*, while samples from patients with squamous cell lung carcinoma (11 patients [37%]) were tested retrospectively. Among patients with squamous cell lung carcinoma, 1 patient was found to have *EGFR* exon 19 deletion and 1 patient was found to have *EML4*-*ALK* rearrangement. Prior to enrollment in the study, patients had already received a median (range) of 2 (2-5) chemotherapy regimens (eTable 3 in Supplement 2). Sixteen patients (53%) had received pemetrexed combined with a platinum-based chemotherapy regimen as first-line therapy. The remaining patients had received platinum-based chemotherapy combined with docetaxel (7 patients [23%]), paclitaxel (4 patients [13%]), or gemcitabine (3 patients [10%]). A total of 3 patients (10%) received bevacizumab in either the first- or second-line setting.

**Table 1.** Baseline Characteristics of Included Patients

Characteristic	No. (%) (N = 30)
Age, median (range), y	63 (34-78)
Sex	
Men	18 (60)
Women	12 (40)
Cigarette smoking status	
None	14 (477)
Former	16 (53)
Histologic type	
Adenocarcinoma	19 (63)
Squamous cell carcinoma	9 (30)
Adenosquamous carcinoma	2 (7)
Cancer stage	
Stage IV	27 (90)
Stage IIIB or IIIC	3 (10)
ECOG performance status score	
0-1	20 (67)
≥2	10 (33)
Brain metastasis	
Yes	2 (7)
No	28 (93)
Driver oncogenes	
* EGFR*	1 (3)^a^
*EML4*-*ALK*	1 (3)^b^
* ROS1*	0
None	28 (93)
Prior chemotherapy regimens received, median (range), No.	2 (2-5)
2	27 (90)
3	1 (3)
4	1 (3)
5	1 (3)
Prior surgical procedure	6 (20)
Prior radiotherapy	3 (10)

Abbreviations: *ALK*, anaplastic lymphoma kinase; ECOG, Eastern Cooperative Oncology Group; *EGFR*, epidermal growth factor receptor; *EML4*, echinoderm microtubule-associated protein-like 4 gene; PS, performance status.

^a^ Patient’s squamous cell lung carcinoma was initially identified as wild type, but retrospective analysis of specimen using next-generation sequencing revealed *EGFR* exon 19 deletion.

^b^ Patient’s squamous cell lung carcinoma was initially identified as wild type, but retrospective analysis of specimen using next-generation sequencing revealed *EML4*-*ALK* fusion.

By December 31, 2019, the data cutoff date, all 30 patients had discontinued the treatment, with a median (interquartile range) follow-up of 11.0 (4.5-14.1) months. Patients received apatinib plus vinorelbine for a median (range) of 4 (1-22) months. Ten patients (33%) received treatment for less than 2 months, 4 patients (13%) received treatment for 2 to 4 months, 7 patients (23%) received treatment for 4 to 6 months, 3 patients (10%) received treatment for 6 to 8 months, and 6 patients (20%) received the treatment for longer than 8 months (eTable 4 in Supplement 2). Most patients (25 patients [83%]) continued the treatment until disease progression, while the remaining 5 patients (17%) discontinued the treatment owing to adverse events.

### Efficacy Assessment

**Figure 1.**
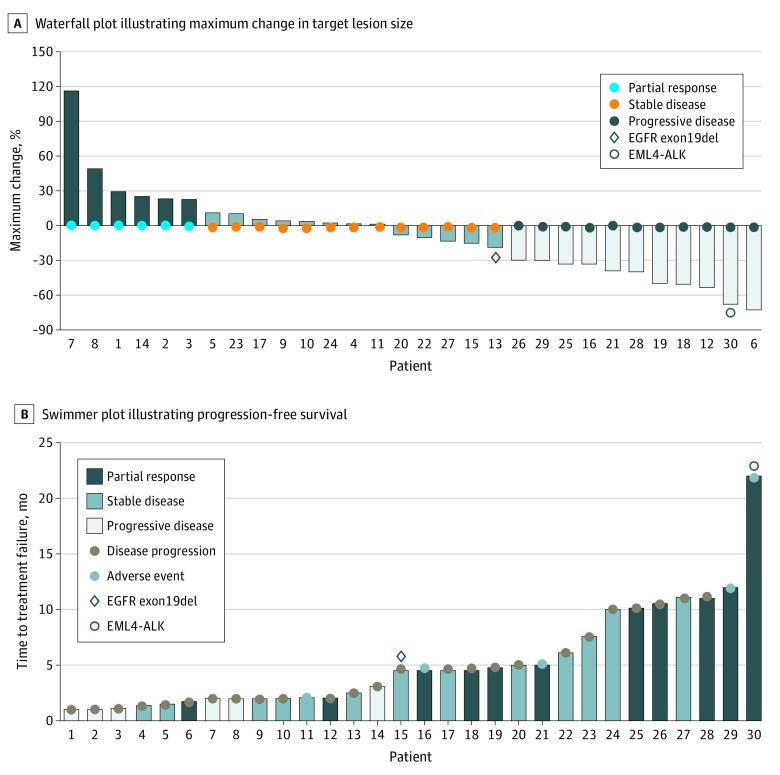
Clinical Outcomes of the Cohort

**Figure 2.**
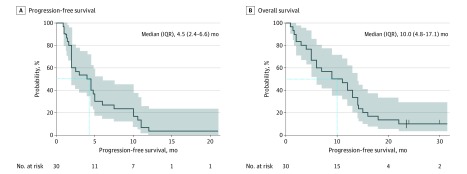
Survival Outcomes of the Cohort Shaded areas indicate 95% CI; dotted line, median survival; and IQR, interquartile range. Tick marks (B) indicate patients who were still alive as of the cutoff date of December 31, 2019.

Table 2 summarizes the response assessments for the cohort. Overall response was achieved in 11 patients (37%). Meanwhile, disease control was achieved in 23 patients (77%), and 7 patients (23%) did not respond to the treatment regimen. Figure 1 illustrates the tumor shrinkage and duration of response. The maximum reduction in size of target lesions from the baseline was 33%. The median PFS was 4.5 (95% CI, 2.4-6.6) months (Figure 2A). Median overall survival was 10.0 (95% CI, 4.8-17.1) months (Figure 2B). The dosage of apatinib had to be reduced for 13 patients (43%) owing to intolerable toxic effects. All patients who had dose reduction only had the apatinib dose reduced once to 250 mg once daily (eTable 4 in Supplement 2). Apatinib doses were adjusted for almost half of the patients who had apatinib dose reduction during or at the completion of the first cycle (6 patients [46%]); 3 patients (23%) had dose reduction during the second cycle, 3 patients (23%) had dose reduction during the third cycle, and 1 patient (8%) had dose reduction during the fourth cycle. No dose adjustment was necessary for oral vinorelbine.

**Table 2.** Objective Response of the Cohort

Group	No. (%)							*P* Value
	Total	CR	PR	SD	PD	DCR	ORR	
Total	30 (100)	0	11 (37)	12 (40)	7 (23)	23 (77)	11 (37)	
Apatinib dose, mg								
500^a^	17 (57)	0	6 (35)	7 (25)	4 (24)	11 (65)	6 (35)	.90
250	13 (43)	0	5 (39)	5 (39)	3 (23)	10 (77)	5 (39)	

Abbreviations: CR, complete response; DCR, disease control rate; ORR, overall response rate; PD, progressive disease; PR, partial response; SD, stable disease.

^a^ Includes all patients who did not require dose reduction.

### Safety

The incidence of adverse events at any grade regardless of causality was 86.7% (eTable 5 in Supplement 2). Most adverse events observed were grade 1 to 2, including hypertension (3 patients [10%]), proteinuria (7 patients [23%]), and hand-foot syndrome (13 patients [43%]). Hand-foot syndrome was the most common adverse event observed, including grade 3 hand-foot syndrome observed in 5 patients (17%) and grade 4 hand-foot observed in 1 patient (3%). Five patients (12%) discontinued the study owing to adverse events, including grade 3 weakness (1 patient [20%]), pleural effusion (1 patient [20%]), fungal infection (1 patient [20%]), and grade 3 hand-foot syndrome (2 patients [40%]). No febrile neutropenia, perforation or fistulation events, or fatal adverse events were observed in our cohort.

## Discussion

The findings of this nonrandomized clinical trial suggest the 3-D coculture model is promising for initiating future clinical trials based on potentially efficacious agents identified from drug susceptibility tests using patient-derived cells grown in 3-D coculture platform. Three-dimensional in vitro cell culture models are more accurate than 2-dimensional (2-D) cell culture models, as well as faster and cheaper than animal models.^21^ Compared with 2-D cell cultures, 3-D cell cultures can preserve the in vivo signaling pathways that are critical in driving tumor cell proliferation, progression, and metastatic spread, which allows a more accurate response to therapeutic agents.

Single-agent apatinib or vinorelbine has been used in the management of NSCLC after failure of prior lines of chemotherapy or targeted therapy.^18^ Based on the clinical and survival outcomes, the results of our study suggest that the combination of apatinib and oral vinorelbine is potentially efficacious in the third-line setting and beyond among patients with variation-negative advanced NSCLC, suggesting its potential as treatment option in this subset of patients. In our cohort, an ORR of 37% was associated with treatment with apatinib combined with oral vinorelbine in the third-line setting. In a trial on apatinib combined with docetaxel in advanced lung adenocarcinoma patients with wild-type *EGFR*,^23^ median PFS was 2.76 months. We hypothesize that the observed extension in progression-free survival or treatment effect was associated with a potential the synergistic effect of both antiangiogenic (apatinib) and antimicrotubule(vinorelbine) drugs.^24^ Since antiangiogenic drugs and antimicrotubule drugs are capable of inhibiting tumor growth as single agents, the use of these drugs in combination may be associated with a synergistic effect, which enhances their inhibitory effect, leading to an improvement in the survival time of patients. Another advantage is that the combination of both drugs is not associated with cumulative adverse effects, as opposed to treatment with doublet cytotoxic chemotherapy. Moreover, this combination therapy has an additional advantage in that both apatinib and vinorelbine were orally administered and did not require hospital admission or an infusion pump, which could improve the adherence of the patient to the treatment regimen, in turn possibly leading to better therapeutic results. In our trial, most experiences of hypertension, proteinuria, and hand-foot syndrome were grade 1 to 2, with incidences of 10% for hypertension, 23% for proteinuria, and 63% for hand-foot syndrome, which were generally consistent with results reported in a 2019 study of apatinib.^25^ All the toxic effects observed in our cohort were manageable and no treatment-related deaths were recorded.

### Limitations

This study has some limitations. Since our study has single-group design, our conclusions are inherently limited by the lack of a control group, and thus selection bias could not be ruled out. Additionally, the proportion of patients who received dose modification was higher than expected.

## Conclusions

This nonrandomized clinical trial found that the combination of apatinib and oral vinorelbine has promising efficacy and manageable toxic effects as a third-line or subsequent-line treatment in patients with driver variation–negative advanced NCSLC. Further evaluation of this combination in phase 3 trials is warranted.

## Notes

Supplement 1. **Trial Protocol**

Supplement 2. **eFigure 1.** Schematic Diagram of the 3-Dimensional Coculture-Based Clinical Trial **eFigure 2.** Efficacy Apatinib and Vinorelbine Guided by 3-Dimensional Coculture Platform **eFigure 3.** Clinical Study Design **eTable 1.** Drugs Included in the Screening Package **eTable 2.** Baseline Characteristics of Patients Whose Tissues Were Used for the 3-Dimensional Coculture Assay For Drug Susceptibility **eTable 3.** Treatment History of Each Patient **eTable 4.** Treatment Duration **eTable 5.** Adverse Events

## References

[1] Siegel RL, Miller KD, Jemal A Cancer statistics, 2018. CA Cancer J Clin. 2018;68(1):-. doi:10.3322/caac.21442 29313949

[2] Feng RM, Zong YN, Cao SM, Xu RH Current cancer situation in China: good or bad news from the 2018 Global Cancer Statistics? Cancer Commun (Lond). 2019;39(1):22. doi:10.1186/s40880-019-0368-6 31030667 PMC6487510

[3] Kedia S, Garcia G, Dhar M Stage IV EGFR mutation-negative and ALK mutation-negative lung adenocarcinoma: long-term survival is possible. Cureus. 2015;7(12):e419. doi:10.7759/cureus.419 26835190 PMC4725754

[4] Nishiyama A, Katakami N, Yoshioka H, Retrospective efficacy and safety analyses of erlotinib, pemetrexed, and docetaxel in EGFR-mutation-negative patients with previously treated advanced non-squamous non-small-cell lung cancer. Lung Cancer. 2015;89(3):301-305. doi:10.1016/j.lungcan.2015.06.017 26141215

[5] Sarosi V, Losonczy G, Francovszky E, Effectiveness of erlotinib treatment in advanced KRAS mutation-negative lung adenocarcinoma patients: results of a multicenter observational cohort study (MOTIVATE). Lung Cancer. 2014;86(1):54-58. doi:10.1016/j.lungcan.2014.07.011 25129367

[6] Rossi A, Chiodini P, Sun JM, Six versus fewer planned cycles of first-line platinum-based chemotherapy for non-small-cell lung cancer: a systematic review and meta-analysis of individual patient data. Lancet Oncol. 2014;15(11):1254-1262. doi:10.1016/S1470-2045(14)70402-4 25232001

[7] Gerber DE, Schiller JH Maintenance chemotherapy for advanced non-small-cell lung cancer: new life for an old idea. J Clin Oncol. 2013;31(8):1009-1020. doi:10.1200/JCO.2012.43.7459 23401441 PMC3589699

[8] Wang Z, Zhang H, Zhou C, Real-world outcomes of various regimens of recombinant human endostatin combined with chemotherapy in non-driver gene mutation advanced non-small cell lung cancer. Cancer Med. 2019;8(4):1434-1441. doi:10.1002/cam4.2014 30762300 PMC6488207

[9] Davies J, Patel M, Gridelli C, de Marinis F, Waterkamp D, McCusker ME Real-world treatment patterns for patients receiving second-line and third-line treatment for advanced non-small cell lung cancer: a systematic review of recently published studies. PLoS One. 2017;12(4):e0175679. doi:10.1371/journal.pone.0175679 28410405 PMC5391942

[10] Kerbel RS Tumor angiogenesis. N Engl J Med. 2008;358(19):2039-2049. doi:10.1056/NEJMra0706596 18463380 PMC4542009

[11] Inoue M, Hager JH, Ferrara N, Gerber HP, Hanahan D VEGF-A has a critical, nonredundant role in angiogenic switching and pancreatic beta cell carcinogenesis. Cancer Cell. 2002;1(2):193-202. doi:10.1016/S1535-6108(02)00031-4 12086877

[12] Ellis LM, Hicklin DJ VEGF-targeted therapy: mechanisms of anti-tumour activity. Nat Rev Cancer. 2008;8(8):579-591. doi:10.1038/nrc2403 18596824

[13] Carmeliet P, De Smet F, Loges S, Mazzone M Branching morphogenesis and antiangiogenesis candidates: tip cells lead the way. Nat Rev Clin Oncol. 2009;6(6):315-326. doi:10.1038/nrclinonc.2009.64 19483738

[14] Fischer C, Mazzone M, Jonckx B, Carmeliet P FLT1 and its ligands VEGFB and PlGF: drug targets for anti-angiogenic therapy? Nat Rev Cancer. 2008;8(12):942-956. doi:10.1038/nrc2524 19029957

[15] Xue JM, Astère M, Zhong MX, Lin H, Shen J, Zhu YX Efficacy and safety of apatinib treatment for gastric cancer, hepatocellular carcinoma and non-small cell lung cancer: a meta-analysis. Onco Targets Ther. 2018;11:6119-6128. doi:10.2147/OTT.S172717 30288047 PMC6160267

[16] Li J, Qin S, Xu J, Apatinib for chemotherapy-refractory advanced metastatic gastric cancer: results from a randomized, placebo-controlled, parallel-arm, phase II trial. J Clin Oncol. 2013;31(26):3219-3225. doi:10.1200/JCO.2013.48.8585 23918952

[17] Roviello G, Polom K, Roviello F, Targeting VEGFR-2 in metastatic gastric cancer: results from a literature-based meta-analysis. Cancer Invest. 2017;35(3):187-194. doi:10.1080/07357907.2016.1276185 28165839

[18] Liu Z, Ou W, Li N, Wang SY Apatinib monotherapy for advanced non-small cell lung cancer after the failure of chemotherapy or other targeted therapy. Thorac Cancer. 2018;9(10):1285-1290. doi:10.1111/1759-7714.12836 30126078 PMC6166085

[19] Altinoz MA, Ozpinar A, Alturfan EE, Elmaci I Vinorelbine’s anti-tumor actions may depend on the mitotic apoptosis, autophagy and inflammation: hypotheses with implications for chemo-immunotherapy of advanced cancers and pediatric gliomas. J Chemother. 2018;30(4):203-212. doi:10.1080/1120009X.2018.1487149 30025492

[20] Kang DH, Park DI, Chung C, Efficacy of weekly vinorelbine monotherapy in patients with lung adenocarcinoma. J Clin Oncol. 2018;36(15)(suppl):e21157-e21157. doi:10.1200/JCO.2018.36.15_suppl.e21157

[21] Fang C, Man YG, Cuttitta F, Novel phenotypic fluorescent three-dimensional co-culture platforms for recapitulating tumor in vivo progression and for personalized therapy. J Cancer. 2013;4(9):755-763. doi:10.7150/jca.7813 24312145 PMC3842444

[22] World Medical Association World Medical Association Declaration of Helsinki: ethical principles for medical research involving human subjects. JAMA. 2013;310(20):2191-2194. doi:10.1001/jama.2013.281053. 24141714

[23] Duan JC, Wang ZJ, Lin L, Apatinib, a novel VEGFR inhibitor plus docetaxel in advanced lung adenocarcinoma patients with wild-type EGFR: a phase I trial. Invest New Drugs. 2019;37(4):731-737. doi:10.1007/s10637-019-00735-1 30706337

[24] Mahaddalkar T, Lopus M From natural products to designer drugs: development and molecular mechanisms action of novel anti-microtubule breast cancer therapeutics. Curr Top Med Chem. 2017;17(22):2559-2568. doi:10.2174/1568026617666170104144240 28056739

[25] Yang C, Feng W, Wu D Apatinib for advanced nonsmall-cell lung cancer: a retrospective case series analysis. J Cancer Res Ther. 2018;14(1):159-162. doi:10.4103/0973-1482.172582 29516980

