# Supplementary material for: Efficacy and Safety of Apatinib Plus Vinorelbine in Patients With Wild-Type Advanced Non–Small Cell Lung Cancer After Second-Line Treatment Failure: A Nonrandomized Clinical Trial
Source: JAMA Netw Open. 2020 Mar 19;3(3):e201226. doi: 10.1001/jamanetworkopen.2020.1226 (PMC7082721; doi:10.1001/jamanetworkopen.2020.1226)
Supplement: Supplement 1. — Trial Protocol [file jamanetwopen-3-e201226-s001.pdf]

Protocol

Main inclusion criteria:

Age≥18，Pathologically identified non-small cell lung cancer

Adenocarcinoma, No-drive gene mutations (with EGFR、ALK、ROS1 included detected by next generation sequencing, NGS)

Squamous cell carcinoma，there is no need to perform gene detection but will be retrospectively detected by NGS

Progression disease after second line treatment

Patients have not been administrated with Vinorelbine or apatinib

PS score ≤2

Estimated survival time more than 3 months

Main exclusion criteria:

Patients received treatment of apatinib or Vinorelbine before

EGFR, ALK or ROS1 mutation positive for Lung Adenocarcinoma patients

EGFR, ALK or ROS1 mutation positive for Squamous cell carcinoma if the patients have performed gene detection by NGS

Patients with contraindication of chemotherapy

Pregnant or breast feeding women

Outcome Measures:

Primary Outcome: Objective Response Rate (ORR)

ORR means the best response status during the whole treatment line.

Secondary Outcome Measures: Progression free survival (PFS), OS (Overall survival) and safety

Sample size calculation:

We have chosen a fixed sample size of 30

Statistical analysis:

SPSS (version 22.0) was used for statistical analyses. Proportion of responders were calculated with the 95% confidence intervals (CI) using the Clopper-Pearson method. Kaplan-Meier method was used to estimate the median duration of response and PFS with corresponding 95% CIs.
