# Supplementary material for: Efficacy and Safety of Apatinib Plus Vinorelbine in Patients With Wild-Type Advanced Non–Small Cell Lung Cancer After Second-Line Treatment Failure: A Nonrandomized Clinical Trial
Source: JAMA Netw Open. 2020 Mar 19;3(3):e201226. doi: 10.1001/jamanetworkopen.2020.1226 (PMC7082721; doi:10.1001/jamanetworkopen.2020.1226)
Supplement: Supplement 2. — eFigure 1. Schematic Diagram of the 3-Dimensional Coculture-Based Clinical Trial eFigure 2. Efficacy Apatinib and Vinorelbine Guided by 3-Dimensional Coculture Platform eFigure 3. Clinical Study Design eTable 1. Drugs Included in the Screening Package eTable 2. Baseline Characteristics of Patients Whose Tissues Were Used for the 3-Dimensional Coculture Assay For Drug Susceptibility eTable 3. Treatment History of Each Patient eTable 4. Treatment Duration eTable 5. Adverse Events [file jamanetwopen-3-e201226-s002.pdf]

Supplementary Online Content

Zhang X, Xiong Y, Xia Q, et al. Efficacy and safety of apatinib plus vinorelbine in patients with wild-type advanced non–small cell lung cancer after second-line treatment failure: a nonrandomized clinical trial. *JAMA Netw Open*. 2020;3(3):e201226. doi:10.1001/jamanetworkopen.2020.1226

eFigure 1. Schematic Diagram of the 3-Dimensional Coculture-Based Clinical Trial
eFigure 2. Efficacy of Apatinib and Vinorelbine Guided by 3-Dimensional Coculture Platform
eFigure 3. Clinical Study Design
eTable 1. Drugs Included in the Screening Package
eTable 2. Baseline Characteristics of Patients Whose Tissues Were Used for the 3-Dimensional Coculture Assay For Drug Susceptibility
eTable 3. Treatment History of Each Patient
eTable 4. Treatment Duration
eTable 5. Adverse Events

This supplementary material has been provided by the authors to give readers additional information about their work.

© 2020 Zhang X et al. *JAMA Network Open.*

eFigure 1. Schematic Diagram of the 3-Dimensional Coculture-Based Clinical Trial

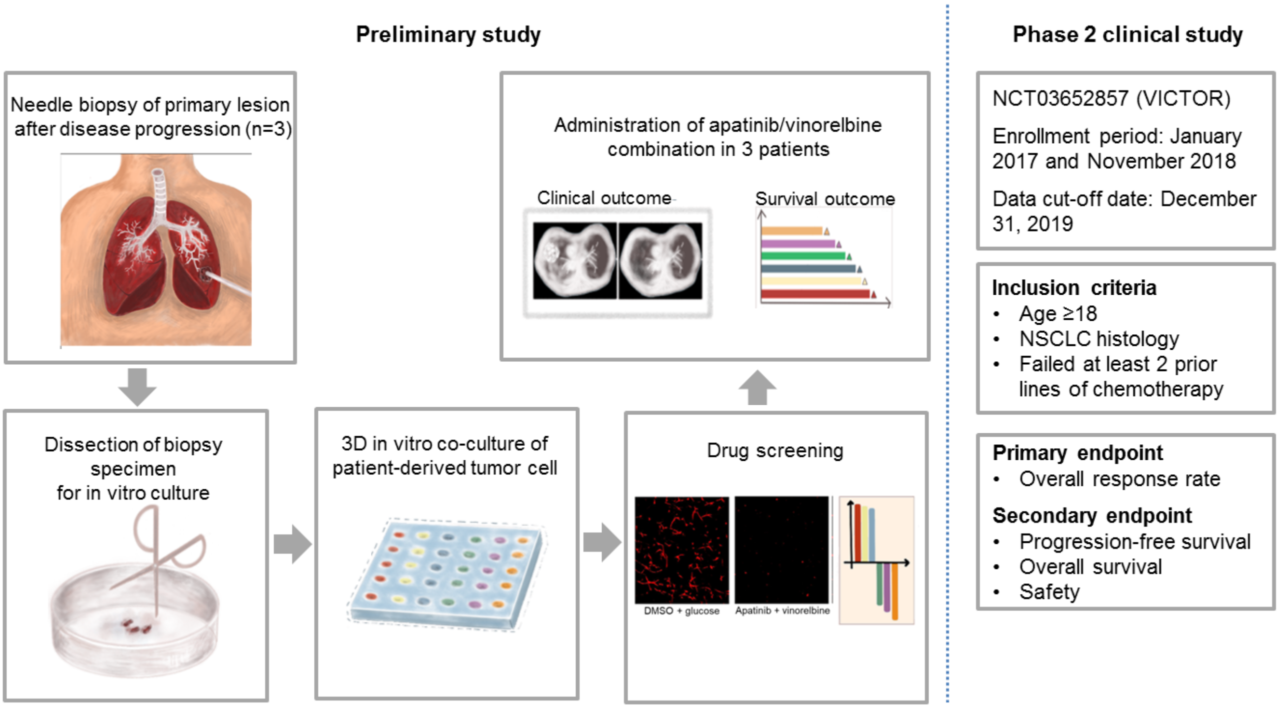

Apatinib and vinorelbine was identified from the 3D co-culture of patient-derived tumor cells obtained from lung tumor biopsy after disease progression. The biopsy specimen was dissected and 3D co-cultured with endothelial cells in culture plate containing growth media. Drug screening was performed using the patient-derived cells treated with indicated concentration of various drugs listed in Table S1. Based on the drug susceptibility test, apatinib and vinorelbine had the most potential in inhibiting the growth of tumor cells and was then administered to the three patients whose tumors were used for the drug screening. The clinical outcomes of the patients were observed prior to initiating the clinical trials for the combination therapy. The clinical trial (NCT03652857) enrolled 30 patients between January 2017 to November 2018 and had overall response rate as the primary endpoint and progression-free survival, overall survival, and safety as the secondary endpoints.

© 2020 Zhang X et al. *JAMA Network Open.*

eFigure 2. Efficacy of Apatinib and Vinorelbine Guided by 3-Dimensional Coculture Platform

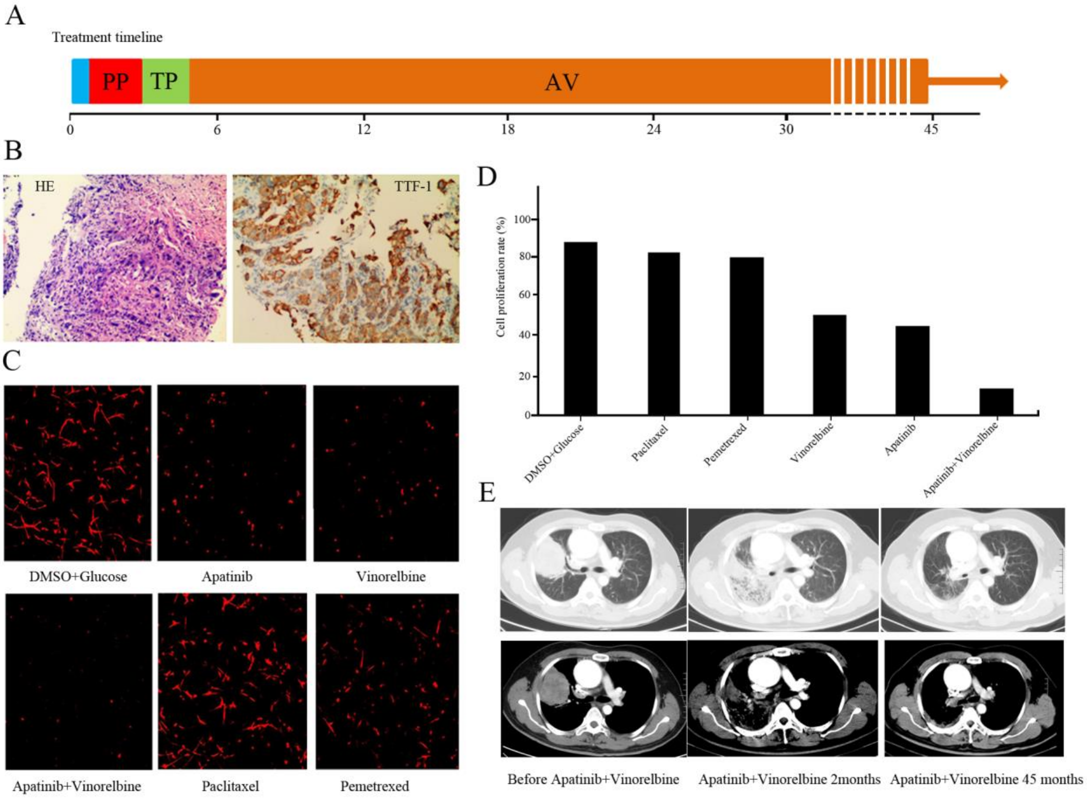

A. Illustration of the treatment timeline received by the male advanced lung adenocarcinoma patient (months). The patient received pemetrexed (PP) and paclitaxel (TP) plus platinum-based chemotherapy doublet regimen and apatinib plus vinorelbine (AV) as first-, second-, and third-line therapy, respectively. B. Micrographs of patient lung biopsy specimen showing the hematoxylin-eosin-staining (left) and immunohistochemistry (TTF-1, right) for histopathologic diagnosis of lung adenocarcinoma. C. Fluorescent micrographs demonstrating the potential of apatinib and vinorelbine combination in inhibiting the proliferation of lung tumor cells derived from the patient as compared to vehicle control (DMSO) or single agent inhibitors including apatinib, vinorelbine, paclitaxel or pemetrexed using the 3D co-culture platform. D. Bar graph illustrating the proliferation rate of patient-derived cells treated with indicated agents. E. CT radiographs demonstrating partial response of the patient to apatinib plus vinorelbine at 2 months from initiating the treatment and sustained response even until 45 months.

© 2020 Zhang X et al. *JAMA Network Open.*

eFigure 3. Clinical Study Design

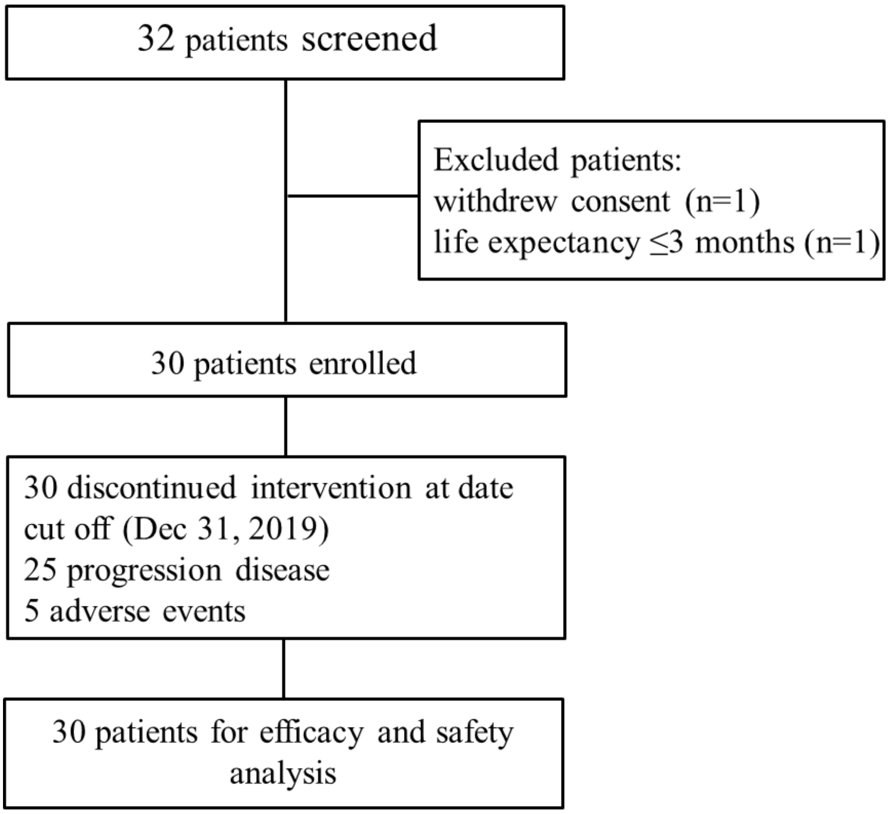

Flow diagram illustrating the clinical study design. A total of 32 patients with advanced non-small cell lung cancer (NSCLC) who were found to have no driver mutations and whose disease progressed from at least 2 lines of chemotherapy. Among them, 2 patients were excluded and 30 patients were included in the study. As of the data cut-off of Dec. 31, 2019; 25 patients discontinued the treatment due to disease progression and 5 patients discontinued due to intolerable adverse events. All patients were evaluated for efficacy and safety.

© 2020 Zhang X et al. *JAMA Network Open.*

eTable 1. Drugs Included in the Screening Package

**Monotherapy**	**Combination therapy**
Vinorelbine	Apatinib and vinorelbine
Cisplatin	Apatinib and docetaxel
Carboplatin	Apatinib and ALIMTA
Gemcitabine	Cisplatin and vinorelbine
Etoposide Injection	Cisplatin and Etoposide Injection
Nimustine	Cisplatin and vinblastinum
Carmustine	Cisplatin and eldisine
ALIMTA	Cisplatin and Gemcitabine
Cyclophosphamide	Cisplatin and Paclitaxel
Glyciphosphoramide	Paclitaxel and Gemcitabine
Paclitaxel	Carboplatin and Paclitaxel
Doxifluridine	Gemcitabine and docetaxel
5-Fluorouracil	Carboplatin and Paclitaxel
Mercaptopurine	
Cytarabine	
Tegafur	
Carmofur	
Hydroxyurea	
Methotrexate	
Ancitabine	
Dactinomycin	
Adriamycin	
Daunorubicin	
Epirubicin	
Mitomycin	
Peplomycin	
Pirarubicin	
Irinotecan	
Harringtonine	
Hydroxycamptothecin	
Taxol	
Vincristine	
Vindesine sulfate	
Vinblastine	
Teniposide	
Atamestane	

© 2020 Zhang X et al. *JAMA Network Open.*

eTable 2. Baseline Characteristics of Patients Whose Tissues Were Used for the 3-Dimensional Coculture Assay For Drug Susceptibility

Patient number	Age (years)	Sex	Smoking history	ECOG PS	Clinical stage	Brain metastasis	Histology	Previous chemotherapy regimen	Driver oncogene mutation	Treatment line of apatinib plus vinorelbine	% maximum tumor reduction	ORR	PFS (months)	OS (months)	Adverse event
1		Male	Former smoker	1	IV	None	Ad	2	Negative	3L	95%	PR	45	Ongoing treatment	Grade I Hand-foot syndrome
2		Male	Former smoker	1	IV	None	Ad	2	Negative	3L	60%	PR	12	26	Grade I Elevated transaminease
3		Male	Former smoker	1	IV	None	Ad	2	Negative	3L	45%	PR	15	27	Grade II Hand-foot syndrome

Abbreviations: ECOG PS, Eastern Cooperative Oncology Group Performance Status; ORR, Objective response rate; PFS, progression-free survival; OS, overall survival; Ad, adenocarcinoma; nL, number of lines

© 2020 Zhang X et al. *JAMA Network Open.*

eTable 3. Treatment History of Each Patient

NO	First-line	Second-line	Third-line	Fourth-line	Fifth-line	Sixth-line	History of surgery	History of radiotherapy
1	Docetaxel+Nedaplatin	Pemetrexed	vinorelbine+Apatinib				Yes	none
2	Pemetrexed+Cisplatin	Docetaxel	Tegafur	vinorelbine+Apatinib			none	none
3	Pemetrexed+Cisplatin	Endostatin+Docetaxel	vinorelbine+Apatinib				none	none
4	Gemcitabine+cisplatin	Endostatin+Paclitaxel+Cisplatin	vinorelbine+Apatinib				none	Yes
5	Pemetrexed+Cisplatin	Docetaxel	vinorelbine+Apatinib				Yes	Yes
6	Gemcitabine+cisplatin	Paclitaxel	vinorelbine+Apatinib				Yes	none
7	Docetaxel+Carboplatin	Icotinib	vinorelbine+Apatinib				none	none
8	Paclitaxel+Carboplatin	Gemcitabine	vinorelbine+Apatinib				none	none
9	Docetaxel+Carboplatin	Gemcitabine	vinorelbine+Apatinib				Yes	none
10	Pemetrexed+Cisplatin	Docetaxel	vinorelbine+Apatinib				none	none
11	Docetaxel+Cisplatin+Endostatin	Pemetrexed+Carboplatin	vinorelbine+Apatinib				none	none
12	Pemetrexed+Nedaplatin	nivolumab	vinorelbine+Apatinib				none	none
13	Paclitaxel+Carboplatin+Endostatin	Docetaxel	vinorelbine+Apatinib				none	Yes
14	Pemetrexed+Carboplatin+Bevacizumab	Docetaxel+Bevacizumab	vinorelbine+Apatinib				none	none
15	Pemetrexed+Cisplatin	Docetaxel	vinorelbine+Apatinib				none	none
16	Pemetrexed+Cisplatin	Paclitaxel+cisplatin	Paclitaxel	Gemcitabine	vinorelbine+Apatinib		none	none
17	Docetaxel+Carboplatin	Pemetrexed	vinorelbine+Apatinib				none	none
18	Pemetrexed+Carboplatin	Docetaxel	vinorelbine+Apatinib				none	none
19	Pemetrexed+Carboplatin	Docetaxel	vinorelbine+Apatinib				Yes	none
20	Gemcitabine+Nedaplatin	Tegafur	vinorelbine+Apatinib				none	none
21	Docetaxel+Carboplatin	Pemetrexed	vinorelbine+Apatinib				none	none

© 2020 Zhang X et al. *JAMA Network Open.*

22	Pemetrexed+Cisplatin	Docetaxel+Bevacizumab	vinorelbine+Apatinib				none	none
23	Pemetrexed+Cisplatin	Docetaxel	Paclitaxel	Gemcitabine	Tegafur	vinorelbine+Apatinib	Yes	none
24	Pemetrexed+Bevacizumab	Docetaxel	vinorelbine+Apatinib				none	none
25	Paclitaxel+Carboplatin	Icotinib	vinorelbine+Apatinib				none	none
26	Paclitaxel+Carboplatin	Docetaxel	vinorelbine+Apatinib				none	none
27	Docetaxel+Cisplatin	erlotinib	vinorelbine+Apatinib				none	none
28	Pemetrexed+Nedaplatin	Gemcitabine	vinorelbine+Apatinib				none	none
29	Pemetrexed+Cisplatin	Docetaxel	vinorelbine+Apatinib				none	none
30	Pemetrexed+Cisplatin	Gemcitabine	vinorelbine+Apatinib				none	none

© 2020 Zhang X et al. *JAMA Network Open.*

eTable 4. Treatment Duration

**Characteristics**	**N=30, N(%)**
**Total duration of treatment cycles,**	4(1-22)
**[median(range), month]**	
**Duration of treatment administration**	
⩾ 8 months	6(20.0%)
⩾6months <8months	3(10.0%)
⩾4months <6months	7(23.3%)
⩾2months <4months	4(13.3%)
<2months	10(33.3%)
**No. of patients with dose reduction**	13(43.3%)
**Reasons for dose reduction (n=13)**	
Grade II-IV Hand-foot syndrome	8(61.5%)
Grade II Leukopenia	1(7.7%)
Grade II Proteinuria	1(7.7%)
Grade II Elevated transaminase#	1(7.7%)*
Grade II Fungal infection	1(7.7%)
Grade III Weakness	1(7.7%)
**No. of patients who discontinued treatment**	30(100%)
**Reasons for treatment discontinuation**	
Grade III Weakness	1(3.3%)
Grade III hand-foot syndrome*	2(6.7%)
Accumulation of pleural effusion	1(3.3%)
Fungal infection	1(3.3%)
Disease progression	25(83.3%)

Note: Hash(#) indicates the patient who still had grade II elevated transaminaseeven after rescue treatment, so following the principal investigator’s advice, the patient reduce the apatinib dose to 250 mg once daily. Asterisk (*) indicates the two patients who still had grade III hand-foot syndrome even after apatinib dose reduction. Hence, both patients discontinued treatment following the principal investigator’s advice.

© 2020 Zhang X et al. *JAMA Network Open.*

eTable 5. Adverse Events

Adverse events	Grade I	Grade II	Grade III	Grade IV	Grade I-IV	Grade III or IV
**Hematologic (n=30)**						
Leukopenia	2(6.7%)	2(6.7%)	0(0%)	0(0%)	4(13.3%)	0(0%)
Neutrophil reduction	1(3.3%)	2(6.7%)	0(0%)	0(0%)	3(10.0%)	0(0%)
Anemia	7(23.3%)	2(6.7%)	0(0%)	0(0%)	9(30.0%)	0(0%)
Thrombocytopenia	0(0%)	2(6.7%)	0(0%)	0(0%)	2(6.7%)	0(0%)
**Non-hematologic (n=30)**						
Hand-foot syndrome	8(26.7%)	5(16.7%)	5(16.7%)	1(3.3%)	19(63.3%)	6(20.0%)
Proteinuria	6(20%)	1(3.3%)	0(0%)	0(0%)	7(23.3%)	0(0%)
Hypertension	2(6.7%)	1(3.3%)	0(0%)	0(0%)	3(10.0%)	0(0%)
Elevated transaminase	14(46.7%)	1(3.3%)	0(0%)	0(0%)	15(50.0%)	0(0%)
Elevated bilirubin	4(13.3%)	1(3.3%)	0(0%)	0(0%)	5(16.7%)	0(0%)
Decreased appetite	9(30.0%)	3(10.0%)	0(0%)	0(0%)	12(40.0%)	0(0%)
Diarrhea	6(20.0%)	6(20.0%)	0(0%)	0(0%)	12(40.0%)	0(0%)

© 2020 Zhang X et al. *JAMA Network Open.*
